# The fundamentals of eye tracking part 3: How to choose an eye tracker

**DOI:** 10.3758/s13428-024-02587-x

**Published:** 2025-01-22

**Authors:** Marcus Nyström, Ignace T. C. Hooge, Roy S. Hessels, Richard Andersson, Dan Witzner Hansen, Roger Johansson, Diederick C. Niehorster

**Affiliations:** 1 Lund University Humanities Lab, Box 201, SE 221 00 Lund, Sweden; 2 Experimental Psychology, Helmholtz Institute, Utrecht University, Utrecht, The Netherlands; 3 Tobii AB, Danderyd, Sweden; 4 IT University of Copenhagen, Copenhagen, Denmark; 5 Department of Psychology, Lund University, Lund, Sweden

**Keywords:** Choosing, Eye tracking, Eye movements, Gaze direction

## Abstract

There is an abundance of commercial and open-source eye trackers available for researchers interested in gaze and eye movements. Which aspects should be considered when choosing an eye tracker? The paper describes what distinguishes different types of eye trackers, their suitability for different types of research questions, and highlights questions researchers should ask themselves to make an informed choice.

## Introduction

This article is the third in a series on the fundamentals of eye tracking (see also Hessels et al., 2025; Hooge et al., n.d.; Niehorster et al., 2025). The articles are aimed at individuals who are (one of) the first in their group, company, or research field to use eye tracking, with a focus on all the decisions one may make in the context of an eye-tracking study. Such individuals may come from academia (e.g., psychology, biology, medicine, educational science, computer science), commercial institutions (e.g., marketing research, usability, decision-making) and non-commercial institutions (e.g., hospitals, air traffic control, military organizations). Note that this is not an exhaustive description of the target audience. More experienced eye-tracking researchers may find useful insights in the article series, or may find the article series a useful reference or hub to relevant research. One may either choose to read this article as the third part of the series, but one may choose to skip the other articles in the series if this article is of more immediate interest.

An eye tracker is an instrument that estimates eye movements or gaze (Holmqvist et al., 2011; Duchowski, 2007; Young & Sheena, 1975; Dunn et al., 2023). Eye tracking has been used for more than a century (Wade & Tatler, 2005) and has led to significant advancements in what we know about human and animal behavior and cognition in several fields, including linguistics, psychology, neuroscience, human factors, and human–computer interaction, to name a few (see also Hessels et al., 2025; Hooge et al., n.d.).

Over the years, a variety of methods have been used to record eye movements using instruments based on many different recording principles. They range from invasive systems that only experts can operate to modern video-based eye trackers that a research assistant can record high-quality data with after a 5-min introduction. Both new and established researchers with an interest in eye movements are at some point in their career likely faced with the question: ‘which eye tracker should I choose?’ In fact, this is one of the most frequent questions we get when giving our eye-tracking courses or when meeting people who are about to start working with eye trackers. The goal of this article is to equip researchers with the knowledge to help answer this question and make an informed choice. This article is relevant both for those who are planning to buy an eye tracker, those who are in the fortunate position to choose from many in their lab, and for those who have an eye tracker but wonder if it is suitable for their next study. Moreover, this article may be useful for researchers who in their grant applications apply for funds to buy an eye tracker. This article is part of a series on the fundamentals of eye tracking. The other articles in the series discuss what questions an eye tracker can help answer (Hessels et al., 2025) and how to operationalize a research question about eye movements (Hooge et al., n.d.). Moreover, Niehorster et al. (2025) provides an overview of tools (besides the eye tracker itself) available to researchers to, for instance, present stimuli, communicate with the eye tracker, and visualize and analyze eye-tracking data.

Is choosing an eye tracker more complicated than simply selecting the eye tracker with the best technical specifications such as sampling frequency, accuracy, and precision? As will be elaborated on in this article, the answer is a resounding yes! Choosing an eye tracker often starts with defining what the requirements are for a particular application or experiment (Valtakari et al., 2021). Clearly, there is a large difference between recording an infant (Franchak, Kretch, Soska, Babcock, & Adolph, (Franchak et al., 2010)), a dog (Park, Holmqvist, Niehorster, Huber, & Virányi, 2023), or adults with their heads stabilized with a bitebar (Steinman, 1996). Since there are so many different requirements, we have given many different answers when colleagues or students ask ‘which eye tracker should I choose’?

We are not the first to address the problem of choosing an eye tracker, and various requirements have previously been highlighted. McConkie (1981) stressed that a very basic eye tracker is sufficient in situations where only a crude estimate of the gaze direction is required, for instance whether participants moved their eyes in the left or right direction. In a similar spirit, Young and Sheena (1975) state that an eye tracker does not necessarily need the best performance to produce useful results, and argue that a system that meets the minimum requirements should be chosen. They further argue that researchers should consider the trade-off between different requirements since some may be more important than others. There are several opinions about what such requirements include. For instance, Sheena and Borah (1981) writes that eye-tracker performance should be maximized by

1. “allowing subjects the maximum possible psychological and physical freedom”.
2. “extracting a large amount of information with the highest possible accuracy”.
3. “enabling successful eye position measurement of the greatest possible subject population (different eye pigmentation, different eyelid shapes and positions, glasses, contact lenses, etc)”.

and Collewijn, Van der Mark, and Jansen (1975) suggest that an ideal eye tracker for precise measurements of eye movements should, among other properties, have “sufficient resolution, linearity and dynamic range (in time and space)” and be able to track participants with little motivation or prior experience of eye tracking. More generally, Valtakari et al. (2021) argue that how to choose an eye tracker always depends on the availability of eye trackers in combination with what requirements it needs to meet, for instance that it is cheap to buy, easy to use, or come with software that supports the desired data processing and analysis needs.

It is clear from the above examples that choosing an eye tracker is more than selecting the one with the best technical specifications. However, such specifications constrain the type of research questions that can be approached. Understanding an eye tracker’s technical properties is therefore critical to be able to make decisions at the design stage of an experiment. By discussing the technical properties of eye trackers in this article, we aim to have the reader make these constraining choices explicitly rather than implicitly being limited by using whatever eye tracker is available.

We begin this article by giving an overview of general considerations that are relevant when choosing an eye tracker, some of which will be elaborated on later in the article. This is followed by a description of different research scenarios, where fictitious researchers known as personas are used to illustrate different needs researchers may have when choosing an eye tracker. We then describe what eye trackers estimate, how they have been classified, and in what research contexts specific properties of eye trackers may be relevant (sections Eye tracker properties and Technical specifications). Finally, examples of how researchers have tested whether an eye tracker meets their particular requirements are presented (Eye tracker evaluation). The article ends with concise advice and a discussion on how to choose an eye tracker. Note that this article is not intended to be a buyer’s guide, and that we intentionally avoid making recommendations about the choice of a specific eye tracker, for instance that if researchers work in field X, they should always choose an eye tracker of model Y. As for many types of decisions, it is difficult to advise on which eye tracker to choose without knowing factors like the needs, expectations, skills, and budget of the researcher. Moreover, we will not argue that some eye tracker properties are more important than others since this depends largely on the specific research context. Critically, the goal is to help readers become better deciders – not decide for them.

There are at least a few ways a reader could approach this article. First, a reader with previous knowledge of eye tracking and familiarity with eye-tracking terminology may want to read the scenarios (Considerations for choosing an eye tracker in specific research contexts) and then how to evaluate eye trackers (Eye tracker evaluation). Second, readers may carefully read only the scenario closest to their own research context, and read the remainder of the article with this scenario in mind. Finally, researchers new to eye tracking will benefit from reading the article from the beginning to the end.

Readers new to eye tracking may find unfamiliar terms in this article, for instance gaze, fixation, and head-free eye-tracker setup. If such terms are encountered, we refer the reader to Table 1, which gives a brief explanation of the terms and points the reader to relevant articles or later sections in the article where the terms are described in more detail.

**Table 1:** A brief summary to eye-tracking terminology used in the article

Term	Brief description	Further reading
Accuracy (deg)	How close the estimated gaze locations are to the true gaze locations.	Accuracy
Area of interest	A delineation of regions inside the stimulus space that can be used to link gaze measures to parts of the visual stimulus.	Hessels, Kemner, van den Boomen, & Hooge (2016)
Calibration	A procedure where participants look at visual targets to adapt to the participant and enable accurate gaze estimation.	Calibration
Corneal reflection (CR)	A reflection of an (generally infrared) external light source on the cornea.	
Data loss	How many samples are reported as invalid or not recorded at all.	Data loss
Eye movement	An eye rotation relative to the head.	Reference frames
Eye tracker	A device to estimate eye movements or gaze.	What is an eye tracker
Eye-tracking data	Anything observable by the use of an eye tracker.	–
Eye-tracker setup	A combination of participant placement and tools (e.g., eye-tracker, screen, tables, chairs, and chinrest) used to record eye-tracking data.	Valtakari et al. (2021)
Fixation	A period of little movement of a gaze point in the world.	Hessels, Niehorster, Nyström, Andersson, & Hooge (2018)
Fovea	A small region in the center of the retina where visual acuity is the highest.	Bringmann and Wiedemann (2021)
Gaze	Where humans look in the world. Can be a direction or a specific location (gaze point).	Reference frames
Head boxed eye-tracker setup	An eye-tracker setup where the participant’s head can move within a frustum (also known as the headbox).	What is an eye tracker
Head-free eye-tracker setup	An eye-tracker setup where the participant’s head can move freely.	What is an eye tracker
Head-restricted eye-tracker setup	An eye-tracker setup where the participant’s head is restricted.	What is an eye tracker
Precision (deg)	How dispersed estimated gaze locations are.	Precision
Saccade	A fast eye movement that directs the fovea toward a new part of the environment.	Hessels et al. (2018)
Sampling frequency (Hz)	How many times per second data are recorded.	Sampling frequency
Validation	A procedure where participants look at fixation targets to evaluate the quality of a calibration.	Calibration

The descriptions of the terminology apply to human participants with normal anatomy and functioning of the visual system. For more information about the terms, we refer the reader to the references under ‘Further reading’

**Table 2:** Examples of requirements of an eye tracker that may be critical for the personas

Persona	Research topic	Main requirements	Type of eye tracker
Boris	Decision-making	Mobility of the setup	Head boxed
Kim	Reading	Accuracy	Head restricted
Ingrid	Human factors	Need to output pupil size	Head restricted
Sven	Cognitive development	Usability, robustness to head movements	Head boxed
Kerstin	Sports research	Robust to slippage and sunlight	Head free
Sam	Covert attention	Precision	Head restricted

### General considerations in choosing an eye tracker

Some general considerations apply to everyone considering choosing an eye tracker, may not be directly related to the intended research question and participant group, and may also apply to instruments other than eye trackers. They include (in no particular order):

- *Life expectancy*. How long is the eye tracker expected to function and be supported? For instance, this may be an important aspect in longitudinal data collections since having to replace an eye tracker in the middle of a study may change the nature of the collected data and thus the outcome measures (De Kloe et al., 2022).
- *Price*. How much does it cost? What is included in the cost (software, support, service)? It may be tempting to buy only the eye-tracker hardware, but that usually means additional costs in terms of time required to develop one’s own custom software.
- *Availability*. Does the researcher have free choice among all available eye trackers, or is the choice limited to one or more eye trackers available at the university/company.
- *Mobility*. How easy is it to pack, transport, and unpack the eye tracker if recordings take place at other locations than a lab, for instance a school or a hospital?
- *Support*. Does the manufacturer provide support? Is the support staff knowledgeable and the support included as part of the eye-tracker purchase? What level of support is provided (advice/hands-on support)? Is there a forum where users of the eye tracker can meet and share problems/experiences? Is the support guaranteed long-term? There are recent examples where big tech companies have bought eye-tracker manufacturers leading to an unexpected and immediate stop of any support, for instance when SensoMotoric Instruments (SMI) was acquired by Apple in 2017.
- *Reputation in the research field.* Certain brands of eye trackers are popular within specific research fields, for instance the EyeLink for reading research (e.g., Bai, Yan, Liversedge, Zang, & Rayner, 2008) and the Tobii TX300 (e.g., Dalrymple, Jiang, Zhao, & Elison, 2019) for infant research. Why is this, and how do researchers in these fields motivate their choices?
- *Maintenance*. Is the eye tracker easy to keep operational, or does it require regular oversight and service? An extreme example is the dual Purkinje eye tracker, which requires regular and sophisticated alignment of optical elements, and consists of moving parts that may suffer from wear and tear (Wu et al., 2023).
- *Power consumption*. This is particularly relevant to wearable systems: can the head-mounted eye tracker record a participant for a whole day without having to charge it? his could be important, for instance, in the context of health monitoring (Vidal, Turner, Bulling, & Gellersen, 2012).
- *Software and tools*. Is there software for stimulus presentation, visualization, and analysis? Does the manufacturer provide a software development kit (SDK)? Is the software backward compatible, such that the same experimental script can be run many years later without modification? Does the eye-tracker setup contain software that can compute relevant measures such as first fixation duration and saccade amplitude, or is that up to the researcher to figure out?
- *Transparency*. Does the manufacturer disclose the main technology and methods used to estimate gaze or is it a black box? As an example of when transparency matters, McConkie (1997) describes a situation where they eventually found out that a filter added a 25-ms delay to the eye-tracking signal, making their gaze-contingent application slower than expected. Are data stored in an open format, or the proprietary format of the manufacturer?
- *Flexibility*. Can the eye-tracker setup be used only for one type of experiment and participant group, or is it possible to adjust the setup to handle many different use cases? For instance, if the research question requires the use of a touch-screen, can participants still be tracked or are the hands and arms blocking the eye-tracker camera’s view of the participant’s eyes?
- *Connectivity and synchronization*. Can the eye tracker interface with external devices such as electroencephalogram (EEG), galvanic skin response (GSR), or motion capture? Does it have a transistor-transistor logic (TTL) input or another method to reliably synchronize eye-tracking data to data from external devices (cf. e.g., Xue, Quan, Li, Yue, & Zhang, 2017)? For a comprehensive overview of tools and advice related to connectivity and synchronization, we refer to the article by Niehorster et al. (2025).

This list can be seen as questions researchers can ask themselves and decide whether they are relevant to their particular research context. We will elaborate on some of these questions later in the article. Other considerations may be directly related to the research question and participant group, for instance:

- *Reference frame*. Does the research question concern where people look in the world (gaze point or direction) or how the eye moves relative to the head (eye movements)? In other words, what frame of reference are data recorded in?
- *Operation*. Is setup and calibration simple and quick? Are there many things an operator can/need to adjust to be able to record certain participants such as infants, elderly, or patients? Do the participants have to sit very still, or even have to be immobilized with a bitebar, chin-, or forehead rest?
- *Binocularity*. Are monocular (from one eye) recordings sufficient, or are binocular (from both eyes) data required?
- *Outcome measure*. What aspects of eye movements and gaze are of interest, for instance fixation location, fixation duration, saccade dynamics, or pupil size?
- *Data quality*. Are the recorded data of sufficient quality to address the research question in terms of, for instance, sampling frequency, latency, accuracy, precision, resolution, or data loss?

As is evident from the above lists, there is a large number of aspects that may play a role when choosing an eye tracker, some of which will constrain the type of research questions that can be approached. Importantly, choosing an eye tracker depends on what kind of requirements need to be met for a specific context (Valtakari et al., 2021). To illustrate such contexts and requirements, we begin this article by considering specific research scenarios.

## Considerations for choosing an eye tracker in specific research contexts

Below, we introduce six personas, i.e., fictitious researchers that may be seen as representative of a specific research context. These personas are used to illustrate considerations a researcher interested in eye movements or gaze may be confronted with when choosing an eye tracker. The personas are selected to represent scenarios that many researchers can relate to, and illustrate research with different eye-tracker setups. The personas also reflect research topics that are familiar to the authors. This selection may be considered arbitrary, and we could have chosen personas representing other relevant research contexts, for instance human interaction (Valtakari et al., 2021), multimedia learning (Van Gog & Scheiter, 2010), or neurological disorders (Leigh & Zee, 1999). Note that while the research contexts may be very different from that of the reader, some of the considerations may overlap. As such, we encourage readers to look beyond the specifics of a research context when judging which personas and considerations are of relevance to them.

The research contexts are intended to briefly illustrate some key aspects that a researcher may need to consider when choosing an eye tracker and are intended to make the reader actively start considering these before reading the remainder of the article. They are not meant to provide a full description of the large number of issues a persona may encounter during the process of designing and executing the study. In this way, the research contexts can be considered as ‘trigger material’ in problem-based learning, which is often used to prompt a discussion and solution to a problem in order to facilitate learning (Wood, 2003). Before reading any further, we encourage the readers to carefully consider these research contexts, and think about and motivate which eye tracker they think each persona should choose. For readers that need a little help in this, the personas along with the research topic, and the main eye tracker requirements that may be relevant in their research contexts are summarized in Table 2. Key references provided in the description of each research context may also be helpful for this purpose.

In the discussion of this article – after the reader may be more familiar with the eye tracker types in Table 2 and various eye tracker properties that may be of relevance – we will revisit these personas and discuss how they may reason when choosing an eye tracker. In the discussion, rather than providing a recommended eye tracker for each persona, we want to model the process of making such a decision, thereby helping the readers ask the right questions and take responsibility for their own choices.

### Decision-making

Boris works in the field of decision-making. Part of his research is founded in the gaze cascade model, which predicts that the proportion of time a participant looks at the preferred option increases prior to a choice response (Shimojo, Simion, Shimojo, & Scheier, 2003). Potential applications of this model include product choice (Atalay, Bodur, & Rasolofoarison, 2012) and making choices in moral dilemmas (Pärnamets et al., 2015). Stimuli are presented on a computer screen where participants can choose between a few options that are large and well separated from each other. He is interested in where participants look, but not how the eye moves during saccades. Boris collects data at companies where he can more easily recruit participants. Moreover, data are often collected with the help of master students or research assistants.

### Reading

Kim is collaborating with the marketing department of a publishing house. They want to know how their (online) journals and newspapers are consumed and can be better adjusted to specific customer groups (see also the section about reading in Hooge et al., n.d.). They have questions like: Do people with a university degree read differently than people without? Do older people read in a similar way as adolescents? Kim has delved into reading research methods and is convinced that a large portion of these questions can be answered with an eye tracker, along with materials and analysis methods from reading research.

### Human factors

Ingrid works at a human factors institute. She wants to test the hypothesis that, either through training or selection, military personnel is more resilient to stress than civilians, as indicated by several subjective and physiological measures (as in Toet, Bijlsma, & Brouwer, 2017). Pupil size, heart rate, and skin conductance were adopted as physiological stress correlates. For pupil size, Ingrid is considering an eye tracker. The human factors institute closely collaborates with the army, thus getting participants to visit her lab is not a problem.

### Cognitive development

Sven is interested in cognitive development during infancy. Sven has worked with multiple observational techniques to investigate infant viewing preferences (e.g., habituation, preferential looking Fantz, 1958; Teller, 1979), but is now considering eye tracking, which may give him more fine-grained insights into infant viewing behavior. He realizes that potential problems for infant eye-tracking research are (1) infants’ unwillingness to be restrained, and subsequent movement in front of the eye tracker, (2) a short attention span, which limits the time available for positioning or calibration, and (3) a lack of comprehension of verbal instructions, which may affect calibration or experimental procedures (Aslin, 2012; Gredebäck, Johnson, & von Hofsten, 2009; Hessels & Hooge, 2019).

### Sports research

Kerstin is a researcher in sports psychology and wants to investigate potential strategies strikers and goalkeepers use during penalty shootouts in football (Wood & Wilson, 2010; Timmis, Turner, & Van Paridon, 2014). She envisions using the eye tracker to determine, for example, whether the striker looks at the ball, goal, or goalkeeper – and at what moments – before taking the shot. While working out the first idea of a study, she finds out that there are no power outlets available on the pitch. She also cannot foresee the weather conditions during the day of the eye-tracking recordings, which could probably be anything from full sunlight to clouds and rain.

### Operationalizing covert attention with microsaccades

Sam is a cognitive neuroscientist with a keen interest in covert attention (see Hooge et al., n.d., for an operationalization). While he has previously studied covert attention indirectly through response time measurements, he is now seeking a method to directly capture the direction in which covert attention is focused. He has read that one way to operationalize the direction of covert attention is by estimating the direction of microsaccades (Engbert & Kliegl, 2003). Microsaccades have been described as small (3–30 min arc) saccades that occur during instructed fixation (Poletti & Rucci, 2016; Nyström, Hansen, Andersson, & Hooge, 2016). The main requirement for Sam is an eye tracker that allows him to estimate when microsaccades occur and in what direction they go.

**Fig. 1:**
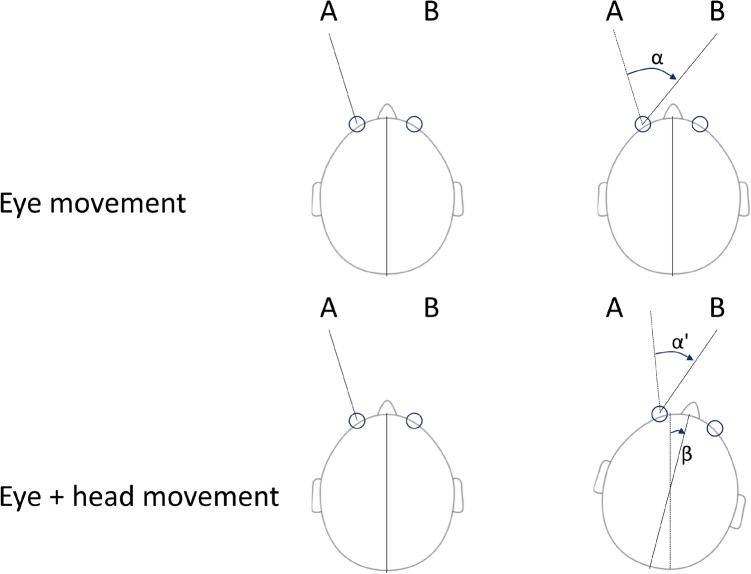
Two examples of how the same gaze movement between two points in the world (A and B) can be achieved. *Top*, by only moving the eye relative to the head (an eye movement, $\alpha$) and, *bottom*, by a combined eye movement ($\alpha ^{\prime }$) and head-in-world ($\beta$) rotation. The gaze movement is a combination of the eye and head movements

## What is an eye tracker and what types are there?

Before choosing an eye tracker, it is advantageous to know what it does, what it consists of, and how it can be implemented. There are several different definitions of an eye tracker. Dunn et al. (2023) write that an eye tracker is an instrument used for “measurement of eye or gaze direction or the movement over time or the parameters derived from the data obtained”, and Morimoto and Mimica (2005) use the term ‘Eye gaze trackers’ that are used to “estimate the direction of gaze of a person”. Duchowski (2007) defines an eye tracker as “The measurement device most often used for measuring eye movements” and, citing Young and Sheena (1975), divides them into two functional classes: “those that measure the position of the eye relative to the head, and those that measure the orientation of the eye in space, or the ‘point of regard’ ”, where the latter term typically refers to the point where the gaze direction intersects with an object in the world, i.e., the gaze point (*cf.* terminology in Table 1). These two classes of eye trackers record data in different frames of reference: head and world. By a world frame of reference, we mean a coordinate system related to the physical world or objects therein, for instance a computer screen, a room, or a football pitch.

The rationale behind the distinction between eye and gaze movements is that it often is of interest to know where people look in the world rather than how the eye is oriented inside the head (Young & Sheena, 1975). For instance, a researcher may want to know whether a participant looks at a specific object in the world, for instance a pasta package on a shelf in a grocery store (Gidlöf, Wallin, Dewhurst, & Holmqvist, 2013), irrespective of whether the participant is standing still relative to the package, or is walking past it. In other words, the researcher is interested in the location in the world looked at, and not in how the eye moves in the head so that gaze is maintained on this location.

In contrast, another researcher may be interested in how the eye counter-rolls relative to the head in response to a head rotation to investigate potential dysfunction of the vestibular system (Halmagyi et al., 2017). Here, it is not of interest to estimate where the participant looks in the world, and investigations can even be performed in complete darkness (Alhabib & Saliba, 2017).

To illustrate the distinction between eye and gaze movements, Fig. 1 shows two different ways to achieve a gaze movement between two fixation points in space (A and B) by 1) moving only the eye relative to the head (top row) or 2) using a combined eye and head movement (bottom row). Note that in 2), the eye movement ($\alpha ^{\prime }$, see Fig. 1) contributes only with part of the whole gaze movement, and the head movement ($\beta$) contributes with the rest. In the remainder of this article, eye movements will refer to eye-in-head movements, whereas gaze movements will refer to changes of the gaze point in the world, irrespective of the exact contribution of eye, head, trunk or body movements with respect to the world (cf. e.g., Land, 2006).

Another way to classify eye trackers is according to their physical components and the tracking principle. There are a number of principles that have been used for eye-, or gaze-movement tracking that are described in detail elsewhere (e.g., Young & Sheena, 1975; Leigh & Zee, 1999; Duchowski, 2007; Hammoud, 2008; Hansen & Ji, 2010; Holmqvist et al., 2011). Broadly, these have been divided into those that use attachment devices such as contact lenses put directly on the eye (e.g., scleral search coils), electro-oculography (EOG) that measures the electrical activity due to eye movements relative to the head, video-oculography (VOG) where eye image features are tracked in video-recordings of the eye (e.g., pupil or Purkinje image tracking, and retinal tracking), and photo-diodes that pick-up reflections of (typically) infrared light from the eye (e.g., limbus tracking).

**Fig. 2:**
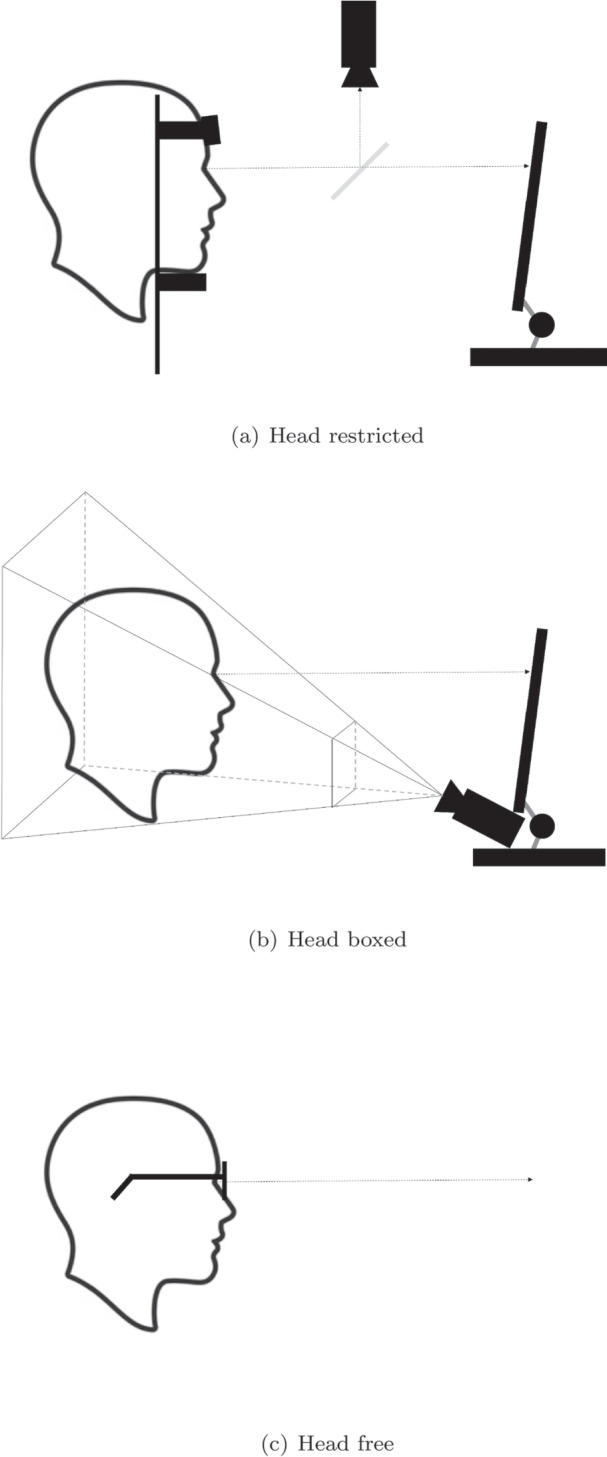
Three types of eye-tracker setups according to Valtakari et al. (2021). (**a**) Head-restricted setup where the eye is filmed through a hot mirror (**b**) head boxed setup where the head is allowed to move within a frustum with respect to the eye tracker, and (**c**) a head-free setup where the eye tracker is mounted on the participant’s head, and the participant is allowed to move freely. In (a) and (b), the stimulus is typically shown on a screen while in (c), the stimulus comprises the world. Note that the head-free setup typically outputs eye movements (eye-in-head orientations), and it is then up to the researcher to map these to objects in the world (e.g., from images captured by the scene camera) during later analysis

Today, the vast majority of eye tracking is video-based (VOG). In this article, we will mainly discuss these and mention explicitly when other techniques are discussed. The minority of researchers who still use attachment devices or other specialized hardware not based on VOG have in our opinion often specific reasons for this choice, for instance to estimate very brief and low-amplitude eye movements (Poletti & Rucci, 2016; Rolfs, 2009) or to compare the performance of newly introduced eye trackers to techniques considered the gold standard in the field (MacDougall, Weber, McGarvie, Halmagyi, & Curthoys, 2009; Van der Geest & Frens, 2002). Moreover, these researchers have often invested heavily in understanding and testing their eye trackers, and are therefore not the target readers for this article.

VOG eye trackers have been classified in different ways. For instance, Holmqvist et al. (2011) refer to tower-mounted, remote, and head-mounted eye trackers. Tower-mount refers to when the camera is mounted close to the eye inside a ‘tower’ to film the eye in high resolution via a hot mirror placed between the participant’s eye and the stimulus. This mirror reflects infrared (IR) light to the camera but allows visible light to pass through, such that participants can view the stimulus through the mirror. An important function of the tower is to stabilize the head relative to the camera using a chin-, and forehead rest. In contrast, with a remote eye tracker, the camera films the eye directly from a distance, and the head is typically free to move within a frustum (also known as the headbox), i.e., a limited volume in front of the eye tracker, outside of which participants typically cannot be tracked successfully. An advantage of using a tower-mounted eye tracker is the freedom to be able to move the hands and arms in-between the screen and the eye tracker (for instance when using a touch screen), without blocking the eye trackers’ view of the eye. Finally, head-mounted eye trackers are attached to the head and follow along when participants move their heads.

**Table 3:** Three types of eye-tracker setups according to Valtakari et al. (2021), along with their frame-of-reference, output, expected data quality, and examples eye trackers and research fields that have been associated with each type

Type	Frame-of-reference	Example output	Data quality	Example field	Example tracker$^a$
Head-restricted	World and head	Pixel on screen	High	Reading, scene perception, oculomotor research	SR Research EyeLink, SMI HiSpeed
Head boxed	World	Pixel on screen	Medium	Infant research, usability, marketing research	Gazepoint GP3, WebGazer, EyeFollower, Tobii TX300
Head free	Head	Pixel in scene camera image	Medium/low	Sports, driving, virtual reality	ASL Mobile Eye , PupilLabs Invisible, Tobii Pro Glasses 3

See Fig. 2 for illustrations of these setups. For more information about the opportunities and challenges with these types of setups, we refer the reader to Hessels, R. S., Cornelissen, T. H., Kemner, C., & Hooge (2015b); Niehorster, Cornelissen, Holmqvist, Hooge, & Hessels (2018, 2021); Fu et al. (2024)

$^a$ For an extensive list of past and currently available eye trackers, see https://wiki.cogain.org/index.php/Eye_Trackers

Using a similar but not identical nomenclature, Valtakari et al. (2021) distinguish between three main types of VOG-eye-tracker setups used in interaction research, where setup refers to the “overall combination of eye tracker and participant” (illustrated in Fig. 2). The first type is referred to as "head-free," where eye tracking is possible with unrestricted head movements, including the use of head-mounted eye trackers. The second type was called head boxed and restricts head movements to a specific volume relative to the eye trackers, similar to a remote eye tracker using the terminology of Holmqvist et al. (2011). A setup in which the participant’s head is restricted with, for instance, head-, or chinrest, a bitebar, or a helmet, is called a head-restricted setup, according to Valtakari et al. (2021).

Do these different types of eye trackers estimate eye or gaze movements? Head-mounted eye trackers typically output eye-movement data in the reference frame of the scene camera, which is attached to the participant’s head. As such, these systems output eye movements (eye orientation relative to the head). Remote and tower-mounted eye trackers typically provide gaze positions relative to the world, for instance a location on a computer screen. If the head is fixed relative to the world, for instance if a remote or head-mounted eye tracker is used with a chinrest, the terms eye and gaze movements can be used interchangeably. After all, when the head is fixed, gaze movements can only be achieved by means of eye movements and any measured gaze movement thus directly reflects movement of the eye inside the head. These terms and issues will be elaborated on later in the article (section  Recording and analyzing in different frames-of-reference).

Importantly, being able to move around comes at a price; head-restricted setups may provide higher data quality in terms of accuracy, precision, and data loss (cf. section Technical specifications) compared to head-boxed and head-free setups. Table 3 provides some examples of what type of research has been conducted and what types of eye trackers have been used in different fields, along with pros and cons of the three types of eye-tracker setups proposed by Valtakari et al. (2021).

VOG-tracking can further be classified on the algorithmic level into 2D feature regression, 3D eye models that recover person-specific parameters such as iris radius and kappa angle, and appearance-based methods (for details, see Cheng, Wang, Bao, & Lu, 2024). A key difference between the first two methods and the appearance-based method is that the latter does not require local image features such as the pupil or corneal reflection to be localized in the eye image, but directly learns a mapping from eye image to gaze using deep learning methods. This has the advantage that highly non-linear mappings between eye images and gaze can be accommodated for, and may lead to more accurate and robust gaze estimation (Cheng et al., 2024). However, these advantages may come at the cost of requiring large training datasets to infer these mappings. Moreover, deep learning methods are often regarded as ‘black boxes’, making it challenging to understand why the gaze estimation produced an inaccurate value or failed completely.

## Eye tracker properties

As seen in the previous section, there are many different types of eye trackers available, each designed to estimate various aspects of gaze behavior. These eye trackers are offered by commercial companies and developed by research groups as open-source projects. So which one should be chosen, and on what grounds should that choice be based? In this section, we discuss eye-tracker properties critical to selecting an eye-tracker setup, including the frame-of-reference in which data are recorded and analyzed, in what situations binocular data may be required, considerations related to calibration of an eye tracker, and in what contexts eye-tracker specifications such as sampling frequency, accuracy, and precision may be important to consider.

### Recording and analyzing in different frames-of-reference

Do the participants need to sit completely still, are they allowed to move their heads slightly, or does the research question require participants to move freely? Is the research question related to eye movements or gaze points in the world? These questions relate to the choice of eye-tracker setup (cf. Fig. 2) and the frame of reference in which eye-tracking data are recorded and analyzed.

Some research questions require eye movements to be recorded from freely moving participants (Rodrigues, Vickers, & Williams, 2002). In such cases, the eye tracker can be mounted on the head of the participant, and data are recorded relative to the head. In many video-based eye trackers, data are recorded as 3-D gaze vectors or 2-D gaze coordinates relative to the eye tracker’s eye-, and/or scene cameras. If only eye movements are of interest, irrespective of what content participants look at, the eye tracker data can be analyzed directly. For example, Dowiasch, Marx, Einhäuser, and Bremmer (2015) recorded participants of varying ages performing two everyday tasks with a head-mounted eye tracker and found that age significantly affected several parameters of saccades such as the frequency, amplitude, and peak velocity. However, it is often of interest to know what object in the world participants look at, and this information is not directly provided by a head-mounted eye tracker. Associating gaze with objects in the world may require tedious manual annotation (Gidlöf et al., 2013; Benjamins, Hessels, & Hooge, 2018), methods that automatically identify and localize objects in the videos (Hessels et al., 2020; Kumari et al., 2021; Niehorster, Hessels, Benjamins, Nyström, & Hooge, 2024), or conducting the analysis in a world where the direction of gaze and the location and orientation of the head and objects are recorded in the same coordinate system (for instance a virtual world, Clay, König, & Koenig, 2019)

In contrast, in a typical head-restricted setup, the participant’s head is fixed relative to a computer screen where a visual stimulus is shown; here, there is a direct mapping between eye-in-head orientations and eye-in-world locations since the head is stabilized in the world. Consequently, any change in gaze direction directly corresponds to a change in eye orientation. This setup is common for studies in, for instance reading (Rayner, 1998), scene perception (Henderson, 2005), and visual search (Najemnik & Geisler, 2005). Unlike head-mounted eye trackers, each gaze point maps uniquely to an object in the world (for now, we assume a static visual stimulus), meaning every time someone looks at a pixel coordinate (*x*, *y*) on the screen, the same image content will be located at this coordinate. Consequently, an analysis of how much time a specific object in the image is looked at is reduced to simply counting how many gaze samples are located within an AOI enclosing this object.

In a head-boxed setup, participants can move their heads freely within a limited volume relative to the eye tracker, which is normally allowed when using a remote eye tracker. Unless the head pose is estimated, it is in fact misleading to talk about ‘remote *eye* trackers’ since they estimate gaze positions in the world (e.g., a screen) rather than eye movements. For instance, consider a participant who fixates a target in the center of the screen and rotates the head side-to-side or up-and-down (Niehorster et al., 2018); even though the eye clearly rotates inside the head, this is ideally not visible in the gaze coordinates recorded in the screen’s (world’s) frame of reference.

Furthermore, a rapid change in gaze direction from one screen location to another could be executed by an eye movement, or a combined eye and head movement (see Fig. 1), without the eye tracker’s gaze output allowing to tell the difference between the two. While the former can be referred to as a saccade, the latter has been described as a gaze shift or gaze saccade (cf. Freedman, 2008; Hooge, Niehorster, Nyström, & Hessels, 2024a). This distinction may seem small, but may be critical when reporting and communicating results about oculomotor behavior. Consequently, a more appropriate name for a ‘remote eye tracker’ would be a ‘remote gaze tracker’ (Hooge et al., 2024a). However, since ‘remote eye tracker’ is already an established term in the field, we will use it in this article. Importantly, since gaze coordinates are recorded in world coordinates (e.g., a screen) with a head-boxed eye tracker, the AOI analysis would still be as simple as in the head-restricted case.

In the head-restricted and head-boxed examples above, we have assumed a fixed world in the form of an image presented on a computer screen. However, the visual stimulus may also move relative to the screen, for example when dynamic visual stimuli such as videos are used. Due to the appearance, disappearance, and displacements of objects in the video, a pixel coordinate (*x*, *y*) on the screen no longer maps to a unique object in the video, making it challenging to count how much time participants spend looking at the objects. Consequently, as with head-mounted eye trackers, it is up to the researcher to establish what object a participant looks at. In the best-case scenario, the location of the visual stimulus can be controlled by the experimenter and is known at all times. In a video recording of a dynamic scene, however, this may not be the case and software supporting dynamic AOIs (Fichtel et al., 2019) or computer vision methods (Larsson, Nyström, Ardö, Åström, & Stridh, 2016; Kumari et al., 2021; Niehorster et al., 2025) may be required to associate gaze coordinates with objects. In contrast to head-mounted eye trackers where each recorded video from the scene camera is unique, such association needs to be performed only once when the same video is shown to all participants.

Some applications require simultaneous eye and head tracking, for instance when testing the vestibular function through video head-impulse testing (v-HIT), where the velocity of the eye and the head are compared during head rotations (Halmagyi et al., 2017), or when observing how eye and head movements are coordinated during large gaze shifts (Freedman, 2008; Hooge et al., 2024a). Head tracking (often limited to rotational velocity and linear accelerations) is available in many of the head-mounted eye trackers discussed above, and external hardware such as inertial measurement units (IMU) may be required (Cognolato, Atzori, & Müller, 2018).

In conclusion, using a head-restricted setup with static stimuli typically makes the analysis easier and less time-consuming. Therefore, if the study design allows it or if the research question can be turned into a design using this combination, it is worth considering.

### Record from one or two eyes?

Eye trackers can be monocular (only one of the eyes is recorded) or binocular (both eyes are recorded). Some eye trackers record from both eyes but deliver only one gaze coordinate (for instance through binocular averaging). Some researchers choose to record only from one of the eyes under the assumption that they move in synchrony and that gaze from the left and the right eyes is directed to similar locations in the world (e.g., a screen) (King, 2011). This assumption is reasonable in healthy participants (but see Collewijn, Erkelens, & Steinman, 1997) and is common when the main research question concerns gaze location in the world, for instance studies about reading, scene perception, and visual search.

In contrast, binocular eye tracking is required to investigate how the eyes move in relation to each other during vergence eye movements that, for instance occur when fixated objects move toward or away from a participant (Jaschinski, 2016) or to monitor how the eyes’ gaze directions align during fixation (Nuthmann & Kliegl, 2009).

There are also good reasons to use a binocular eye tracker besides studying binocular eye movements. For instance, Elbaum, Wagner, and Botzer (2017) compared the accuracy of the dominant, non-dominant, and the binocular average (Cyclopean eye) for participants producing smooth pursuit eye movements by following a moving target. They found that the distance between the moving target and the gaze location reported by the eye tracker was the shortest for the Cyclopean eye (shorter by about 6 arc min) compared to the dominant and non-dominant eyes. Similarly, Hooge, Holleman, Haukes, and Hessels (2019a) found that most participants had a higher accuracy when averaging gaze signals from the two eyes. However, they also emphasized that, for about 1/3rd of the participants, the accuracy was higher when using a single eye instead of the binocular average, and argue that one should evaluate the accuracy for all three types of signals (left eye, right eye, and binocular average), and select the signal that provides the highest accuracy.

Recording binocular data may also be beneficial in terms of data loss and precision. For instance, if data from one eye is missing, there is an option to analyze data recorded from the other eye. Moreover, averaging data from the two eyes may be used to increase the precision (reduce the noise), irrespective of whether it concerns gaze position or pupil diameter. In general, the reduction in noise through averaging is proportional to the square root of the number of averaged signals (Dempster, 2001). Consequently, in the case of a binocular signal with two signals (from the left and right eyes), noise would be reduced by a factor $\sqrt{2}$.

Besides improving the accuracy and precision of gaze signals, binocular eye-tracking data have shown to be useful to separate recording artifacts from small amplitude saccades (microsaccades). Since eye movements are considered to be conjugate (appear in both eyes, see Nyström, Andersson, Niehorster, & Hooge, 2017), a useful way to reduce false positives in microsaccade detection is to only consider microsaccades that occur in both eyes. Engbert and Kliegl (2003), for instance, used a temporal overlap criterion in their microsaccade detection algorithm, such that microsaccades detected in the right and the left eyes needed a minimal overlap in time to be considered for further analysis.

When using a monocular eye tracker, the researcher needs to decide which eye to record from. Several decision rules have been used, including to record from the dominant eye (Wendt, Brand, & Kollmeier, 2014), the left eye (Sogo, 2013), or the right eye (Reichle, Reineberg, & Schooler, 2010), many times without providing a specific motivation why this particular eye was used. Does it matter which eye is chosen? Nyström, Andersson, Holmqvist, and van de Weijer (2013a) found that accuracy was significantly higher (offset was about 0.03 deg lower) in the dominant eye compared to the non-dominant eye. In absolute terms, however, this reduction in error is so small that it is questionable whether it has any significance in most practical applications.

### Calibration and its validation

Generally, people are good in providing a crude estimate of the direction of other’s gaze (Balsdon & Clifford, 2018). However, observing the eye is not enough to accurately estimate the gaze direction since there is a difference between where the eye (and most importantly the pupil) is pointing and where someone is looking (Schaeffel, 2002). Moreover, since this difference is idiosyncratic and the *exact* gaze direction does not correspond to any physiological feature of the eye, it is likely impossible to accurately estimate where someone looks without participant cooperation (Putnam et al., 2005; Wilk et al., 2017; Reiniger, Domdei, Holz, & Harmening, 2021; Kilpeläinen, Putnam, Ratnam, & Roorda, 2021). Therefore, eye trackers are typically calibrated prior to a recording (cf. Niehorster et al., 2025, for an overview of methods for calibrating). During a calibration, the participant is asked to look at a number of targets (typically 1–13) with known locations, for instance a 2D position on a screen or a 3D location in the world. This allows locations of features in the eye image (e.g., the pupil, CR, or P-CR vector) to be mapped to target locations in the world (e.g., a screen Stampe, 1993) and/or individual parameters in an eye model to be estimated (e.g., corneal curvature, Hansen and Ji, 2010; Barsingerhorn, Boonstra, & Goossens, 2017; Liu, Chi, Yang, & Yin, 2022). For some eye trackers, participants are required to keep their heads still during calibration. The proportion of participants that can be successfully calibrated with an eye tracker along with the usability and speed with which a calibration can be conducted may be important considerations when choosing an eye tracker.

A critical assumption for performing a calibration is that participants are capable and willing to accurately fixate the calibration targets. Given this assumption, it may be difficult to calibrate an eye tracker for certain participant groups, such as infants (Hessels, Andersson, Hooge, Nyström, & Kemner, 2015a) or animals (Niehorster et al., 2025), who do not understand a verbal or written instruction to fixate the displayed calibration targets. Here, methods are instead used to cue the attention of the participant to the location of the target, for instance through motion and speech cues, or by attracting their attention with toys (Gredebäck et al., 2009). Other participants who may be problematic to calibrate include people who can understand the instructions, but cannot hold their gaze still on a target or keep a stable head pose. Examples are patients with nystagmus, a condition that makes the eyes continuously oscillate back and forth, thereby preventing a stable fixation (Rosengren, Nyström, Hammar, & Stridh, 2020; Leigh and Zee, 1999, Ch.10).

There are also eye trackers that do not require individual calibration, where gaze direction is inferred directly (Guestrin & Eizenman, 2006; Tonsen, Baumann, & Dierkes, 2020). Importantly, while calibration-free eye trackers may be accurate on average with respect to a whole population, individual gaze estimates may contain significant inaccuracies. For instance, Tonsen et al. (2020) report an average accuracy of 4$^{\circ }$ over a sample of 367 participants. However, as long as eye movements, and not gaze directions, are of interest, such inaccuracies may be unproblematic, as long as they are consistent over the oculomotor range; then eye-movement metrics like saccade amplitude are not affected. Due to the potentially large inaccuracies in individual recordings, calibration-free eye trackers sometimes provide the option to shift the calibration using a one-point offset correction, where the participant is asked to fixate one location in the world whereafter the gaze cursor in the scene camera is aligned to correspond with this location. Note that we distinguish between calibration and offset correction since the latter only shifts the data, whereas the former can include more complex transformations of the data such as scaling, rotation, and shear (Stampe, 1993).

Depending on the eye tracker, performing a calibration once at the beginning of an experiment may not be sufficient to maintain a high enough accuracy over the course of the whole recording. One situation that may motivate a recalibration or an offset correction is that the eye translates relative to the camera. For some head-mounted eye trackers, this may happen when the eye tracker slips on the head (Bernard, Anne-Catherine, & Eric, 2007; Niehorster et al., 2020b). Similarly, in head-restricted setups, if a participant for some reason moves the head too much relative to the chin-, and forehead rest, or temporarily removes the head from it, a recalibration may be necessary. In contrast, head-boxed eye trackers are by design robust to changes in head position and orientation relative to the eye tracker camera, and participants may therefore even take breaks and resume the experiment later without recalibration (for an example, see, e.g., Jostrup et al., 2024).

In general, there may be other reasons to perform an offset correction or a complete re-calibration than slippage of the eye tracker or translation of the participant relative to the camera. For instance, the pupil size may change over the course of an experiment, leading to inaccuracies, due to an effect known as the pupil size artifact (PSA Wyatt, 2010; Wildenmann & Schaeffel, 2013; Choe, Blake, & Lee, 2016; Hooge, Niehorster, Hessels, Cleveland, & Nyström, 2021). Due to the PSA, changes in pupil size may lead to apparent gaze shifts in the eye-tracking signal even though the eyeball does not rotate. Such apparent gaze shifts can be in the order of several degrees (Drewes, Zhu, Hu, & Hu, 2014), and may render it problematic to study vergence eye movements (Jaschinski, 2016; Hooge, Hessels, & Nyström, 2019b). To reduce the effect of PSA on accuracy, it is important that the ambient light conditions during calibration match those during measurement, which can normally be arranged indoors, but may be a challenge outdoors.

As teachers of eye-tracking courses, we often get the question: was the calibration good enough? Directly after a calibration, another set of points is typically shown to the participant with the purpose of evaluating how successful the calibration was. The success is often measured in terms of accuracy, precision, and the amount of data loss (Holmqvist, Nyström, & Mulvey, 2012; Niehorster, Andersson, & Nyström, 2020a, cf. Section Technical specifications). Most of the time ‘good’ refers to accuracy (precision and data loss will be discussed in later sections). There are at least two answers to that question: First, what is ‘good enough’ in the context of the study design? If the question concerns whether the participants looked at one of two large and well-separated areas on a computer screen, the accuracy is not likely a problem for any eye tracker. Moreover, good accuracy may be required only in part of the screen, for instance, in the center if that is where the stimulus is placed. However, if the research question concern on what letter a saccade lands in a text with small font size, perhaps no eye tracker is good enough. Second, most screen-based commercial eye-trackers have accuracies around 0.5 deg (Hooge et al., 2025), which means that this may be a number to aim for, at least for healthy and cooperative adult participants. If the accuracy is much worse, it could be that the participants did not follow the instructions (look at the targets), or that the geometry of the setup has to be adapted, for instance to work around glasses that block the view of the eyes. Another option may be that participants have a visual or neurological disorder that prevents them from fixating the calibration targets accurately.

In terms of accuracy, providing clear instructions to the participants and letting them test the calibration procedure a few times, may be more important than having an eye tracker with good specifications (low accuracy value in the manufacturer’s manual). In addition to providing clear instructions, letting the participants rather than the eye-tracker’s software decide when they are fixating a calibration target, for instance by pressing a button, may also increase the accuracy of a calibration (Nyström et al., 2013a; Poletti & Rucci, 2016). In summary, besides choosing an eye tracker capable of delivering data with sufficient accuracy, it is worth developing skills in how to properly instruct participants during a calibration.

A prerequisite to be able to estimate binocular fixation disparity (the distance between the gaze point of the left and the right eyes during binocular fixation) is that the gaze direction of both eyes is accurately estimated. Švede, A., Treija, E., Jaschinski, W., and Krūmiņa (2015) argue that monocular calibration is required to estimate physiologically plausible fixation disparity values (see also discussion in Nuthmann & Kliegl, 2009; Liversedge, White, Findlay, & Rayner, 2006). Unlike a binocular calibration, where the participant looks at each calibration target with both eyes in a single run, in a monocular calibration, each eye is calibrated separately, while the other eye is occluded. Note that not all eye trackers support monocular calibration, and researchers therefore should be careful to check that this feature is available when choosing an eye tracker if they require it.

### Technical specifications

The technical specifications of an eye tracker may constrain the type of research questions that can be approached. When selecting an eye tracker, a simple but often too naive decision rule could be to look at the specification sheets from all eye-tracker manufacturers and choose the one with best values (e.g., the highest accuracy, precision, and sampling frequency). What type of specifications do manufacturers typically list and what do they mean? In the following, we list commonly reported specifications along with how they are defined, their typical values, and in what situations they are important to consider.

#### Sampling frequency

**Fig. 3:**
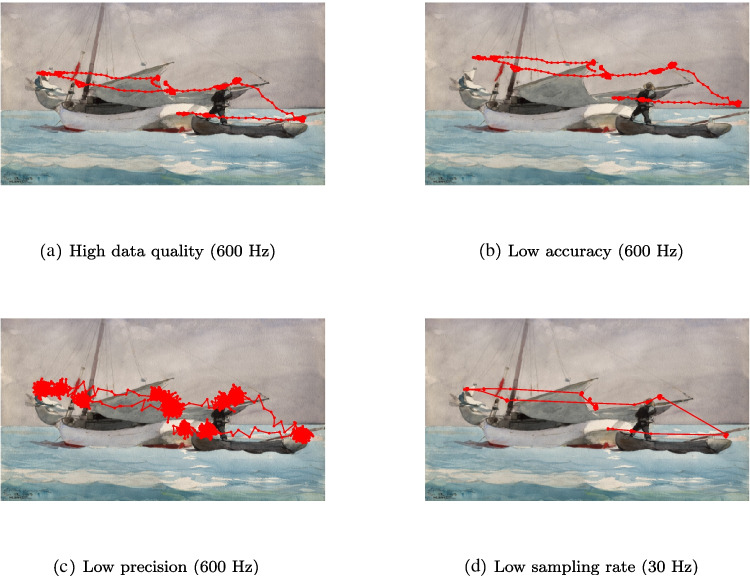
An eye-tracker signal with high data quality in terms of accuracy, precision and sampling frequency (**a**). In the other figures, the accuracy (**b**), the precision (**c**), or the sampling frequency (**d**) was manipulated to be lower than in (a). In (a), the gaze location is close to likely fixation targets, hence the signal has higher accuracy than in (b), where the estimated gaze location is further away from such targets (e.g., people and boats) since data have been shifted upwards and to the right. The precision is higher in (a) since samples around fixations point are more clustered, and lower in (c) since samples are more spread out around fixation points. *Dots* represent where the gaze location is sampled by the eye tracker. A higher sampling frequency means that more samples are recorded per second. The low sampling frequency in (d) prevents detailed investigation of the dynamic properties of the saccades (e.g., peak velocity and waveform) since the gaze position is not sampled during most of the duration of a saccade (note that the lines representing saccades contain very few samples (dots). Data in (a) were recorded with the Tobii Pro Spectrum at 600 Hz from a participant looking at the painting Stowing Sail by Winslow Homer presented on a computer screen for 5 s. The painting spanned 45$\times$26 degrees of visual angle

The sampling frequency (also known as temporal resolution or sampling rate) is a measure of how many times per second data are recorded (Dunn et al., 2023). A sample can contain various information about, for instance, the time the sample was acquired, where gaze is directed, and the pupil size. Eye trackers have sampling frequencies anywhere from 30 Hz (e.g., Skovsgaard, Agustin, Johansen, Hansen, & Tall, 2011) to 2000 Hz (e.g., Schweitzer and Rolfs, 2020). Figure 3 shows a gaze-position signal with high (a) and low (d) sampling frequency. Note, in particular, the lack of samples during saccades in (d) compared to (a).

When is a low sampling frequency of, say, 30 Hz sufficient? Atkins, Tien, Khan, Meneghetti, & Zheng (2013) argued that 30 Hz is enough to investigate what objects surgeons look at in an operating room. A 30-Hz sampling rate provides an estimate of where someone looks every 33.3 ms. It is known that fixations normally last longer than 100 ms, so three samples located close to each other and an object on the display would be enough to assume that the surgeon fixated the object. In general, when researchers are interested in whether participants fixated certain objects in a scene, which may be the most common use of an eye tracker, 30 Hz is sufficient. Examples include sports research (Kredel, Vater, Klostermann, & Hossner, 2017), such as studying the gaze behavior of skilled dart players (Vickers, Rodrigues, & Edworthy, 2000). A low sampling frequency is also sufficient for studies of pupil dilation (Dalmaijer, 2014; Othman & Romli, 2016), due to the rather slow pupil size changes. Can we go even lower, say 1 Hz? In most cases no, given that the eye normally moves 3–5 times per second (Fischer & Weber, 1993), a 1-Hz eye tracker would not accurately estimate fixated locations.

In what type of applications is a high sampling frequency required? The short answer is: when eye movements and in particular saccades and fast gaze shifts are of interest. In terms of accurately estimating saccade peak velocities, previous work has suggested sampling frequencies of at least 200–300 Hz (Inchingolo & Spanio, 1985; Juhola et al., 1985). Below 120 Hz, saccade peak velocities become lower than expected (Mack, Belfanti, & Schwarz, 2017), even though some authors claim that 50 Hz is enough, given appropriate upsampling strategies of the original data (Wierts, Janssen, & Kingma, 2008).

The Shannon–Nyquist sampling theorem provides partial insight into what sampling frequency is required. This theorem states which sampling frequency is sufficient to capture all information in continuous time signals with a finite bandwidth (Shannon, 1949). More precisely, any continuous time signal that contains only frequencies below *x* Hz, can be accurately represented as a discrete time signal if samples are separated by less than $\frac{1}{2x}$ seconds. Since eye movements typically contain frequencies below 100 Hz (Findlay, 1971; Nyström, Niehorster, Andersson, & Hooge, 2021), the minimal sampling frequency required to represent them without information loss would be 200 Hz according to the sampling theorem.

If 200–300 Hz is sufficient to faithfully represent eye movements and estimate saccade peak velocities, why do researchers use even higher sampling frequencies, like 1000 Hz or more (e.g., Ko, Snodderly, & Poletti, 2016)? There are several reasons. First, using a higher sampling frequency can increase the statistical power of an experimental design since more dense sampling reduces the uncertainty of when a certain eye movement behavior started and stopped (Andersson, Nyström, & Holmqvist, 2010). For example, using a 1000-Hz eye tracker reduces the uncertainty of when a saccade begins compared to using a 20-Hz eye tracker since the potential error may be up to 1 ms at 1000 Hz and up to 50 ms in the 20-Hz eye tracker.

Second, using a high sampling frequency may be helpful for early prediction of saccade landing position. In this case, being able to incorporate more samples around the time of the saccade onset in a model increases the likelihood to accurately predict the trajectory of the saccade and therefore its landing position (Han, Saunders, Woods, & Luo, 2013). This is particularly useful in gaze-contingent applications where the visual stimulus needs to be updated as quickly as possible once the position of the saccade landing point is estimated. Moreover, some authors use a very high sampling frequency (e.g., Kimble, Cappello, & Fleming, 2023 who used a 2000-Hz EyeLink), but do not motivate why such a high sampling frequency is required for their particular research question. Finally, Collewijn (2001) used a sampling frequency of 10,000 Hz with a scleral search coil setup and motivates his choice as follows:

To reliably record timing differences on the order of 1 ms between two similarly filtered signals (that have no energy above 100 Hz) it is still necessary to use a sampling frequency that offers the adequate resolution for such a difference. The choice of 10,000 Hz followed from the general rule that the resolution of a measurement should preferably be an order of magnitude better than the expected effect.

Collewijn (2001) chose the high sampling frequency to reliably find very small ($<1$ ms) differences between the onsets of saccades in the left and the right eyes. The 10,000 Hz in combination with a very high precision of his recordings enabled him to discern such differences in single saccades without averaging. In general, according to the law of large numbers, similar sensitivity to detect such small temporal differences could be found using an eye tracker with a lower sampling frequency, given that each participant performs more saccades (see also Andersson et al., 2010), and the differences are independent and identically distributed.

Having a high sampling frequency may be important in situations where eye tracking is combined with other research techniques, such as EEG, to be able to properly align signals from the two techniques (e.g. saccade onset in relation to a electrophysiological signal Dimigen, Sommer, Hohlfeld, Jacobs, & Kliegl, 2011; Degno, Loberg, & Liversedge, 2021). Finally, on a practical note, recording at a high sampling frequency generates more data and therefore requires more storage and may lead to slower data processing.

#### Accuracy

Accuracy, or systematic error, refers to the distance between the true gaze position and the estimated gaze position given by the eye tracker (Holmqvist et al., 2011; Niehorster, Zemblys, Beelders, & Holmqvist, 2020c; BIPM et al., 2012). Since the true gaze position is unknown, accuracy is typically operationalized as the distance between a target the participant is asked to fixate and the gaze position reported by the eye tracker. Instead of providing an accuracy value for each tested fixation target, accuracy is often given as an average across all fixated target positions, participants, and eyes, for instance, “The accuracy was $0.72~\text {deg}~(SD=0.55)$”. Note that this number may not be representative for different positions in the calibrated area, and is typically smaller (better) in the center of the calibration range (Holmqvist et al., 2011).

The reported accuracy of eye trackers range from a few minutes of arc (Poletti & Rucci, 2016) to several degrees (Holmqvist et al., 2011). Accuracy is intimately related to the calibration (Section Calibration and its validation) since the degree to which a high accuracy is achieved is related to how well the participants can follow the instruction to look at points during the calibration and validation procedures.

Most video-based eye trackers that use the pupil center typically do not have accuracies better than 0.5 deg (Hooge et al., 2025; Holmqvist et al., 2011), likely at least in part because of the pupil size artifact (PSA, *cf.* Section Calibration and its validation). When a higher degree of accuracy is required, typically other types of more specialized eye trackers are employed. High accuracy is important when small AOIs are used or when AOIs are spaced closely together, for instance when it is of interest to know the saccade landing position within a word during reading (Radach & McConkie, 1998), or during gaze contingent presentation of simulated scotomas (Geringswald, Baumgartner, & Pollmann, 2013). In contrast, low accuracy may be sufficient in situations where a low number of large and well-separated AOIs are used, for instance in food packaging choice experiments (van der Laan, Hooge, De Ridder, Viergever, & Smeets, 2015), preferential looking experiments (Baillargeon, Spelke, & Wasserman, 1985), or studies using the Visual World Paradigm (Salverda & Tanenhaus, 2017).

#### Precision

Precision refers to the degree to which repeated measurements yield the same results (Poletti & Rucci, 2016), and is commonly operationalized by the root-mean-square of sample-to-sample (RMS-S2S), the standard deviation (SD, Blignaut & Beelders 2012; Niehorster et al., 2020c), or the bivariate contour ellipse area (BCEA, Crossland, Culham, & Rubin, 2004). Precision is intimately related to the noise level in the eye-tracker signal, and these terms are sometimes used interchangeably. Sources of imprecision/noise can include, at least, the measurement device and participant behavior (Niehorster, Zemblys, & Holmqvist, 2021).

Data with low-, and high precision are exemplified in Fig. 3. Compared to accuracy, precision values vary more across different VOG eye trackers. According to Holmqvist et al. (2011), RMS-S2S precision varies between $0.01-0.05^{\circ }$ for tower-mounted eye trackers and $0.03-1.03^{\circ }$ for remote eye trackers. For head-mounted eye trackers, precision has been reported to be in the range $0.09-0.35^{\circ }$ (Hooge, Niehorster, Hessels, Benjamins, & Nyström, 2023).

High precision is required to resolve small amplitude eye movements that otherwise may be obscured by noise in the eye-tracker signal (Collewijn & Kowler, 2008; Poletti & Rucci, 2016), and reduces the need for signal processing (e.g., filtering) when classifying raw data samples into eye-movement events such as fixations and saccades (Nyström & Holmqvist, 2010). High precision may also be beneficial in human-computer interaction when gaze is used to, for instance click a button (Špakov, 2012; Feit et al., 2017).

There are situations where low precision may be sufficient. For instance, when a researcher who wants to know whether a participant looked inside one of two large, well-separated areas of interests, which is the case for, for instance, a preferential looking experiment (Teller, 1979) or an antisaccade task (Everling, Spantekow, Krappmann, & Flohr, 1998), or for a researcher who is interested in fixations and uses a fixation classifier specifically developed to deal with low precision data (Hessels, Niehorster, Kemner, & Hooge, 2017).

#### Data loss

According to Holmqvist et al. (2012), data loss refers to data that are reported as invalid by the eye tracker. Importantly, the same sample may contain both invalid and valid data depending on which data stream is considered. For instance, the pupil size may be valid whereas the gaze position is not. Data loss may occur if the participant blinks, is out of range from the eye tracker, or if tracking fails for other reasons such as mascara that interferes with eye image feature detection, or droopy eyelids that occlude the pupil (Nyström et al., 2013a; Blignaut & Wium, 2014). If the majority of data loss is due to blinks, the pupil size signal can be used to estimate blink parameters such as rate and duration (Pedrotti, Lei, Dzaack, & Rötting, 2011). Otherwise, additional techniques such as eyelid tracking may be required to separate blinks from data loss (Nyström, Andersson, Niehorster, Hessels, & Hooge, 2024) The proportion of lost samples can be operationalized as the number of invalid samples divided by all samples reported by the eye tracker. However, Hooge et al. (2023) experienced a problem with this operationalization when analyzing data from different head-mounted eye trackers since data loss according to the operationalization did not take into account samples that were not reported at all by the eye tracker. For instance, if an eye tracker has a reported sampling frequency of 100 Hz, but only reports 90 valid samples (and no invalid samples) during a 1-s recording, the proportion of data loss is 0, when in fact 10% of the data are missing. To resolve this issue, Hooge et al. (2023) proposed the measure *effective frequency*, operationalized as the number of valid samples divided by the duration these samples were recorded. In the example above, the effective frequency would be 90 Hz since 90 samples were recorded in 1 s. Since samples sometimes may not be reported by the eye tracker and since some eye trackers have variable sampling frequencies (Hessels et al., 2015b; Liu et al., 2018a; Hooge et al., 2023), it is important that researchers compute and report sampling frequency from data recorded in their own experiments. Reporting empirically estimated values instead of manufacturer reported values is crucial for issues like replication, and understanding the data and the results of a study.

Blinks occur about 20 times per minute and last between 150 and 400 ms (Stern, Walrath, & Goldstein, 1984; Nyström et al., 2024). Consequently, up to about 10% data loss can be expected only due to blinks. With remote eye trackers, the data loss also depend on where in the head box participants are located (Nyström et al., 2024) and how much they move (Hessels et al., 2015b; Niehorster et al., 2018).

Data loss may influence measures such as the number of fixations, fixation duration, number of blinks, and saccade rates (Holmqvist et al., 2012). Therefore, if one expects that a certain participant group or condition would produce a larger amount of data loss, this may need to be considered in the analysis (Hessels & Hooge, 2019).

There are also situations where data loss is expected to be less problematic. Consider a researcher interested in relative differences in how much participants look at different objects in a visual stimulus; as long as the amount of data loss is consistent across time and gaze directions, data loss will, on average, not influence such relative differences.

#### Other specifications

Sampling frequency, accuracy, precision, and data loss are arguably the most commonly reported specifications of eye trackers and are relevant in many research contexts. However, there are other properties that may be relevant when targeting specific research questions, and that researchers may want to evaluate themselves. These include the ability to track a range of participants (e.g., with varying age, eye physiology, or visual aids) under various recording conditions (e.g., different level of light, when participants move, and look in eccentric gaze directions). Some applications, for instance those involving gaze-contingent stimulus manipulation (Loschky & Wolverton, 2007; Aguilar & Castet, 2011) benefit from timely delivery of eye-tracker data, and require data to be available with low latency (Holmqvist et al., 2012; Reingold, 2014). Very specific properties like the linearity, sensitivity and resolution are sometimes used to describe the quality of an eye tracker. Here, we refer the interested reader to Poletti and Rucci (2016), who provide an overview on methods to study microsaccades.

## An eye-tracking setup is more than the eye tracker

An eye tracker is much more than its technical specifications, and only one part to consider when choosing an eye-tracker setup. The setup can, for instance, include a computer with selected hardware, software, a screen, a chair, a table, and means to mount the eye tracker relative to the room or the head (Hessels & Hooge, 2019), and the physical conditions of the recording environment (e.g., the light level). In this section, we will discuss such considerations related to how the eye tracker is intended to be used. For a more comprehensive list of tools available to facilitate research with eye-tracker setups, see part 4 in this article series (Niehorster et al., 2025) where, in particular, the sections Hardware and Software below are elaborated on.

### Hardware

An eye-tracker setup can comprise many different types of hardware, including that internal to the eye-tracker or stimulus computer (e.g., processor, RAM, USB, LAN/WLAN, and hard drives) and external hardware such as screens, keyboards, and mice. Turning eye images into eye-tracking data is a demanding tasks for a computer, in particular when real-time delivery of data is required. Factors such as processing demands or bottlenecks in data transfer can slow down or change the expected sampling frequency of an eye tracker, and some eye-tracker manufacturers report sampling frequency as a range instead of a fixed number (Hooge et al., 2023).

In many research scenarios, for instance the visual world paradigm (Salverda & Tanenhaus, 2017), experiments include presentation of both visual and auditory stimuli. The quality and timing of such stimulation depend critically on aspects of the hardware setup, for instance screens, graphics and audio cards. Evaluation of the timing of visual stimulus presentation in relation to the eye-tracking data may be a crucial step when choosing an eye-tracking setup. Tools like The Black Box ToolKit^1^ and StimTracker^2^ may be helpful for this purpose (see also Niehorster et al., 2025, p. 16).

Similarly, a computer with a powerful processor and graphics card may be necessary to be able to use many open and corporate software packages for offline data visualization and analysis.

### Software

Software is typically required to 1) present stimuli on screens/projectors or in VR headsets, 2) communicate with the eye tracker (start, stop, and request data from, Dalmaijer, Mathôt, & Van der Stigchel, 2014; Niehorster et al., 2020a), and for 3) data visualization and analysis. Data processing and analysis may include everything from data ‘cleaning’ (Eskenazi, 2024), to detecting fixation or saccades (Salvucci & Goldberg, 2000; Nyström, Hooge, & Holmqvist, 2013b; Hessels et al., 2017), as well as computing the total fixation time on an object in a recording from a head-mounted eye tracker (Duchowski, Peysakhovich, & Krejtz, 2020).

When acquiring an eye tracker, one or all three functionalities may be provided in the included software. Generally, using manufacturer software is a good choice for researchers using simple experimental designs or standard paradigms, where common options for stimulus presentation and analysis may already be implemented. Examples include presentation of static images accompanied by AOI-analysis (Lazarov, Abend, & Bar-Haim, 2016), presenting text and extracting common reading eye-movement measures (Mézière, Yu, Reichle, Von Der Malsburg, & McArthur, 2023). More custom solutions are typically required for less conventional experimental designs, for instance gaze contingent research (Loschky & Wolverton, 2007), where stimuli may need to be manipulated and updated in real time. Here, it may be useful to adopt programming-based tools developed for stimulus presentation (Peirce, 2007; Peirce, 2008; Brainard, 1997; Mathôt, Schreij, & Theeuwes, 2012) and communication with the eye tracker (Dalmaijer et al., 2014; Niehorster & Nyström, 2020; Niehorster et al., 2020a, 2025).

The availability and functionality of suitable software tools may be important aspects to consider when choosing an eye tracker. For instance, the choice of software tools for data analysis may influence the outcome of a study (Dörzapf, Peitek, Wyrich, & Apel, 2024). Moreover, Dowiasch, Wolf, & Bremmer (2020) found that, to achieve consistent results, applying a standardized analysis of eye-tracking data, for instance for the purpose of saccade detection, was often more important than which eye tracker the data were recorded with. More generally, they argue that the choice of analysis software may be more important than the choice of eye tracker. For a comprehensive overview of software tools available for eye tracking and considerations for choosing the appropriate tool (e.g., for fixation classification Andersson, Larsson, Holmqvist, Stridh, & Nyström, 2017; Hooge, Niehorster, Nyström, Andersson, & Hessels, 2022), we refer the reader to Niehorster et al. (2025).

### Cost

Eye trackers cost anywhere from the price of an ordinary webcam to several thousand euros. For instance, the popular EyeTribe remote eye tracker was long sold for $99 (e.g., Dalmaijer, 2014). The cost for an open-source eye tracker typically originates from two sources: the hardware cost, and costs associated with assembling and testing the eye tracker. For commercial eye trackers, additional costs including factors such as research and development and customer support, are added. Part of the cost could be proprietary software for stimulus presentation and data visualization and analysis.

Funke et al. (2016) tested both more expensive and cheaper eye trackers (<$1000) and found that data recorded from eye trackers on the cheaper end contained more data loss compared to the more expensive ones. They also found that accuracy and, perhaps surprisingly, precision were comparable across the low-, and high-cost eye trackers. Similarly, Dalmaijer (2014) found the EyeTribe eye tracker to lose more data than the much more expensive EyeLink 1000 tracker, but that both eye trackers provided similar results in terms of fixation measures and pupillometry. Due to the lower sampling frequency, however, the EyeTribe eye tracker was deemed unsuitable to study detailed properties of saccades (such as velocity and acceleration). Dalmaijer (2014) emphasize that this comparison was conducted under optimal conditions. Co-recording data from an EyeTribe and a much more expensive eye tracker from SensiMotoric Instruments (SMI), Titz, Scholz, and Sedlmeier (2018) found that both gaze position and pupil size values were strongly correlated between the eye trackers, and concluded that cheaper eye trackers like the EyeTribe may provide results similar to more expensive ones under certain conditions. In general, however, choosing a cheap eye tracker may come at the cost of a worse tracking performance in terms of for instance lower precision, higher amount of data loss, lower ability to calibrate various participants, lower robustness to head movements, and higher risk of having a variable sampling frequency.

Buying a cheap eye tracker may however be a costly investment in the long run, in particular for researchers with no or little computer skills or familiarity with programming. This point is nicely summarized by Titz et al. (2018) who emphasize that there are other costs than the bare eye tracker such as time to set up the eye tracker, developing software for data collection and analysis, and making sense of the raw data. Given what a researcher costs per hour, buying an already existing and fully working solution, including support, may therefore be a more cost-efficient solution. As also pointed out by Titz et al. (2018), it is likely that this situation may change in the future, as increasingly more free software packages become available that help researchers with data collection and/or analyses that do not require advanced programming knowledge. Such open-source initiatives include for instance PsychoPy (Peirce, 2007, 2008), Titta (Niehorster, Siu, & Li, 2015), PyGaze (Dalmaijer et al., 2014), OpenSesame (Mathôt et al., 2012), GazeAlyze (Berger, Winkels, Lischke, & Höppner, 2012), and GraFIX (Saez de Urabain, Johnson, & Smith, 2015).

### Head stabilization

Head-restriction by means of e.g., chin-, and forehead rest, is common practice in many types of studies, for instance in reading research. Why do researchers choose to restrict their participants? There are many potential reasons: First, some eye trackers require participants to have stabilized heads by design. For instance, commercial eye trackers like the EyeLink 1000 from SR Research requires head stabilization when used in tower or desktop mode. Moreover, there is a direct trade-off in how stabilized the head is and the quality of the recorded eye-tracker data (Young & Sheena, 1975; Hermens, 2015; Vienola, Holmes, Glasso, & Rossi, 2024). Therefore, great measures have been taken to stabilize the head. For instance, Wu et al. (2023), using the DPI eye tracker, stabilized the head using both a bitebar and a tight-fitting helmet to minimize artifacts in the eye-tracker signal due to small head movements, to allow the recording of minute microsaccades. Artifacts from head movement may themselves be indistinguishable from waveforms in the eye-tracker signal originating from genuine microsaccades (Hermens, 2015; Holmqvist, Örbom, & Zemblys, 2022). Second, as previously mentioned, head stabilization may be necessary to study eye (in head) movements since with a non-stationary head, the eye-tracker signal may comprise a combination of eye and head motion. Third, head stabilization is routinely used to maintain a fixed distance between the participant’s eye and the visual stimulus, which is important when the angular size of a stimulus needs to be constant on the retina. Finally, using a chinrest removes the need for head-tracking in cases where the head position either needs to be controlled or measured (Halverson & Hornof, 2001).

There are different ways to achieve head restriction, for instance through the already mentioned chinrest and forehead rest as well as bite-bars and helmets. There are also more custom solutions. For instance, Wahl, Dragneva, and Rifai (2019) provide the following description in their methods section (cf. their Figure 1A):

The subject’s head was placed in the chin and forehead rest with foam cushions on the inner sides for a stable head support. Thereafter, the subject’s head was fixed by an elastic band tightened around the forehead rest and the subject’s head to reduce the head movements to a minimum.

Other head-restriction methods include to continuously monitor the position of the head, and alert the participant when the deviation from the desired position becomes too large (Li, Joo, Yeatman, & Reinecke, 2020). For instance, using camera-based monitoring of the head (Kaduk, Goeke, Finger, & König, 2024) write:

At the start of the study, the subject is allowed to select a comfortable head position. Throughout the study, the subject’s head movements are continuously monitored, and the trial is automatically interrupted if the subject moves away from the initial head position. Unlike a physical chinrest, the virtual version provides on-screen feedback and instructions.

Whether interrupting a trial in this manner is acceptable is up to the individual researcher to decide.

So far, reasons why head restriction is required or beneficial have been given, but what are the potential drawbacks? A common argument against using head-restriction we frequently hear from students in our courses is that participants are not able to behave as they normally would, which in turn could influence the gaze behavior (cf. Holleman, Hooge, Kemner, & Hessels, 2020, for a discussion about how the terms naturality and ecological validity are used in psychological science). Even though earlier work has found that saccades were faster and shorter when recorded with a free head compared to a bite-bar (Collewijn, Steinman, Erkelens, Pizlo, & Van Der Steen, 1992), we have not been able to find any reports on whether the now more commonly used chin-, and forehead rest has any influence on eye movements or gaze behavior in research contexts where participants sit in front of a screen. If a researcher has no reason to assume that using a chinrest would influence participants’ gaze behavior in a way meaningful for their study, using head-restriction may thus be motivated from a data quality perspective. A potential drawback of using a chinrest has been discussed in an experiment where eye tracking was combined with arm movements. Here, the authors raise the concern that using a chinrest may “interfere with naturalistic reaching conditions” (Halverson & Hornof, 2001). In general, whether using a chinrest influences the gaze behavior is an empirical question that researchers may wish to address if they think it is problematic for their study design.

To what extent head stabilization is achieved is not only related to the device used (e.g., bite bar or chin rest), but also how the device is mounted to the world and how comfortably and stably it is positioned. As such, both chairs and tables may have a significant impact on the quality of the recorded eye-tracking data (for more detail on these issues see Niehorster et al., 2025).

It is important to mention that head movements may play an important role under certain circumstances, such as during visual exploration in specific tasks. For instance, Franchak, McGee, and Blanch (2021) showed that the contribution of the head to gaze shifts depends on whether the participants search or walk in an outdoor environment.

## Eye tracker evaluation

We highly recommended testing the eye tracker of interest in a setting specific to the intended experiment. What could such testing include? There are at least two different ways eye trackers have been evaluated: 1) how close is the performance to a state-of-the-art or another, already established eye tracker and 2) how well does the tested eye tracker deal with a known or expected behavior. In the first category, there is a plethora of studies comparing eye trackers during different tasks, including looking at static or moving dots on a computer screen. This is a good option if the researcher is already using an eye tracker and is considering replacing it with a newer model (e.g., De Kloe et al., 2022) or if there is an established standard in the field that many researchers accept and trust (Deubel & Bridgeman, 1995; Houben, Goumans, & van der Steen, 2006; Dalmaijer, 2014; Ehinger, Groß, Ibs, & König, 2019; Nyström et al., 2021; McCamy et al., 2015). When possible, such comparisons are conducted by simultaneous co-recording of the eye trackers for the same participants and tasks, such that the signals can be compared directly. However, such co-recording is not always possible since the eye trackers may interfere with each other’s operation, as was the case in for instance Nyström et al. (2021). There are also studies that report comparisons between two or a number of selected eye trackers (Ooms, Dupont, Lapon, & Popelka, 2015; Funke et al., 2016).

The following paragraphs contain examples of the second way to evaluate eye trackers, i.e., how well the eye tracker deals with a known or expected behavior. In one example, Hessels et al. (2015b) and Niehorster et al. (2018) were interested in choosing a remote eye tracker best suited for infant research, and wanted to test how such eye trackers deal with different types of head and body movements expected from such a participant group. Ideally, they would recruit infants and ask them to elicit the behavior that was expected to be problematic for the eye trackers. For obvious reasons, this was impossible. Instead, they let adult participants mimic infant behavior, including looking towards and away from the screen and thereby orienting the head away from the eye-tracker’s camera. Testing a number of different remote eye trackers, they found that performance in terms of accuracy and data loss differed significantly across eye trackers and recommended researchers to test their own setups.

Other researchers may be interested in questions such as ‘can gaze to the nose, mouth, and eye areas be accurately captured with a head-mounted eye tracker during a live conversation when participants are free to move, talk, and may (accidentally) adjust the position of the eye tracker on the head?’ For example, Niehorster et al. (2020b) found that some of the tested eye trackers were sensitive to slippage and became less accurate by several degrees only due to talking or making facial expressions. To address a similar question with a later generation of head-mounted eye-trackers, Hooge et al. (2023) had participants stand still, walk, skip, and jump while looking at a target fixed in space. Since this task was easy for the participants to perform, they expected the estimated gaze direction from the eye tracker to intersect with the visual target. Consequently, deviations between the target and the estimated gaze position could be attributed to inaccuracies in the eye-tracker data. Hooge et al. (2023) found that the eye trackers they tested were mostly robust to head and body movements (most offsets between the target and estimated gaze positions were below 3$^\circ$ during the tasks), which is good news for researchers interesting in for instance sports research.

Atkins et al. (2013) evaluated eye trackers in the context of a surgical environment. They provided three research scenarios and recommended an eye tracker for each scenario. For investigating how surgeons look at objects in an operating room, they recommend a head-mounted eye tracker, and found a sampling frequency of 30 Hz to be sufficient. However, this eye tracker was not found to be accurate enough to reveal where on the surgical display monitor the surgeons looked. Therefore, they first tested a head-boxed eye tracker with a 17-inch computer screen. While this eye tracker was accurate enough, its operating distance (camera to participant) was too short to be used in a real operating room where the distance between the surgical display monitor and the surgeon was longer. Their solution was to employ another head-boxed eye tracker that could be detached from the screen (see Figure 3 in Atkins et al., 2013). This is an illustrative example of how a particular research scenario demands specific requirements of an eye tracker.

Since it is impossible to precisely control the orientation and movements of human eyes, artificial eyes have been used to evaluate eye trackers. Typically, artificial eyes are used to answer questions such as ‘what are the smallest eye movements that can be reliably detected in the estimated gaze signal?’ and ‘does the eye tracker output reproducible gaze signals?’ (Crane & Steele, 1985; Reingold, 2014; Holmqvist & Blignaut, 2020). In such evaluations, the artificial eyes are mounted on motors that precisely rotate the eyes. The main disadvantage of using artificial eyes is that they are not real, and it is therefore unclear whether eye-tracker signals recorded from artificial eyes are representative of signals recorded from real eyes (Wang, Mulvey, Pelz, & Holmqvist, 2017; Niehorster et al., 2020c).

One way to think about an eye-tracker test is to consider the best case scenario: having experienced participants, an experienced operator, and record the data in a highly controlled environment. If the data do not fulfill the requirements of the experiment in this best case scenario, it is unlikely that data collected during a more representative situation would. If data do meet the requirements, tests with the intended target population could be conducted to address questions like how glasses and head movements influence the desired outcome measures. If a researcher is concerned about specific experimental situations, these can be tested to explore the nature of potential error sources and assess their magnitudes.

What can researchers do if they perform an evaluation of their eye tracker and find it is not suitable for their research context? One option would be to choose another eye tracker. Critically, it may also be possible to rethink the design of the experiment and make it more robust to the problems the evaluation revealed; for instance increase the size of an object or the spacing between objects in case the accuracy turned out to be too low due to, for instance participant movement.

## Discussion

The goal of this article is to help researchers ask the right, critical questions and make an informed choice when choosing an evaluating an eye tracker. Importantly, this includes not only the eye tracker but the whole eye-tracker setup, which can include how the participant is placed in relation to the eye tracker (Valtakari et al., 2021) and tools available to support the desired data analysis. Besides discussing relevant requirements that may be considered for this choice, we have provided example research scenarios that we hope will spark the reader’s understanding of what parameters choosing an eye-tracker setup may entail. Note that this article is not a buyer’s guide. Instead, when considering what eye tracker to chose, we advise readers to:

1. Let the research context and questions guide the choice of eye tracker.
2. Evaluate the eye tracker and test whether it is suitable to conduct the intended research.
3. Consider the whole eye-tracker setup, and not only the eye tracker.
4. Discuss the choice of eye tracker with researchers who are working in similar fields.

Specific research contexts may be associated with certain requirements that the eye tracker must fulfill, for instance that the eye tracker must be able to record data outside where sunlight may affect the eye image quality or a newly recruited student must be able to operate the eye tracker after a 5-min introduction. If the research question concerns whether participants looked at the left or right side of the screen, most eye trackers will suffice, for instance web-camera based eye trackers (Semmelmann & Weigelt, 2018; Kaduk et al., 2024) or iPads (Taore, Tiang, & Dakin, 2024). In contrast, other research questions may require information about where someone looks with high accuracy (Ko, Poletti, & Rucci, 2010), high sampling frequency (Collewijn, 2001), or high tolerance to head movements (Hessels et al., 2015a).

Given that some properties are more important than others in a given context, choosing an eye tracker typically involves trade-offs. There are many trade-offs that may be relevant to consider. For example, placing the participant in a chinrest or using a bitebar restricts the degree of head movement and may introduce some discomfort, but generally make it easier to accurately and precisely estimate eye-, and gaze movements (Young & Sheena, 1975). Whether head restriction influences gaze behavior is an open question, but empirical evidence supporting such a speculation seems to be lacking (cf. Eye tracker properties).

Another trade-off concerns the degree of freedom to adjust the eye tracker versus its ease-of-use. For instance, some eye trackers offer various options to fine-tune the hardware (e.g., the focus of the camera or change the location and angle of the camera relative to the eye) and software (e.g., change a threshold to detect the pupil center or limit the area in the eye image where the pupil may be found) to be able to accommodate recordings of a wide range of participants in different conditions. Other eye trackers can successfully track most participants out-of-the-box, but if there is a problem, there is not much the researcher can do to solve it since few options to adjust the eye tracker are available (cf. e.g., Nyström et al., 2021). The adjustable eye tracker may be useful in situations where the number of available participants is small, for instance in certain patient groups, and it is critical that eye-tracker data are successfully recorded for all participants. In contrast, the easy-to-use eye tracker typically allows for a quick setup by operators with little training and, if for some reason a participant cannot be tracked, a new one can easily be recruited and recorded instead.

Moreover, many researchers may be limited by a budget and, like many other products, you may ‘get what you pay for’. More expensive eye trackers may produce data with more information (e.g., a higher sampling frequency or an estimate of eyelid position) higher quality (e.g., precision), and decrease the amount of work the researcher needs to invest to have a fully functional setup (e.g., by coming bundled with software for data processing and analysis). We would argue that a disproportionate amount of attention is paid to the price of the eye tracker, when in fact the cost of salaries for research staff required to, for instance developing software for stimulus presentation and data analysis is typically much higher. It is worth to note, however, that a more expensive eye tracker does not necessarily improve all aspects of data quality; in video-based eye trackers that use the pupil center to estimate gaze, the pupil size artifact (PSA) seems to be one factor why such eye trackers generally do not output data with accuracies better than 0.5 deg (Hooge et al., 2025).

Sometimes, the research context is not known beforehand. This may be the situation in larger labs where a lab manager or professor needs to choose one or several eye trackers to be used for an unforeseeable future. This is the case in the Lund University Humanities Lab, where two of the authors of this article work, and where the eye-tracking facilities are open to all researchers at Lund University. Here, we have optimized on maximal flexibility by acquiring different types of eye trackers that cover most of the scenarios in this article (cf. Considerations forchoosingan eye tracker in specific research contexts), i.e., a combination of head-free, head-boxed, and head-restricted setups with varying degrees of mobility and price range. In addition, to allow research about more in-depth questions about eye-tracking technology and methodology, we also use custom setups with high-resolution, high-framerate cameras and in-house developed image processing and gaze estimation pipelines (Hooge et al., 2021; Nyström, Niehorster, Andersson, Hessels, & Hooge, 2023; Hooge et al., 2024a).

We would advise readers to talk to other researchers in their field before choosing an eye tracker. These researchers may not only know about the strength and weaknesses of a particular eye-tracker setup, but may also be willing to share software, procedures to evaluate the eye tracker, or forward questions to which they do not know the answer to colleagues within their networks.

Finally, which eye tracker is suitable for the intended research may depend on the skills of the individual researcher, and the whole research team. Researchers knowledgeable in for instance experiment design, programming, statistical analysis, hardware, and electronics may be more flexible in their choice compared to researchers who rely on a specific manufacturer software package (further elaborated on in Niehorster et al., 2025).

### How did the personas reason when choosing an eye tracker?

In the beginning of this article, personas were used to represent researchers from a wide array of research contexts with different needs. After reading this article, we hope these personas would be able to motivate their choice of an eye-tracker setup(for examples of what eye tracker types and models could be suitable for the personas’ needs, *cf.* Tables 2 and 3). What are the minimal requirements for an eye-tracker setup these personas need?

Boris, working in decision-making, needs an eye tracker that is mobile, which can easily be packed, transported, and unpacked. Moreover, Boris wants the eye tracker to be robust to wear and tear since it will be frequently used and transported. A high accuracy and precision may not be required since his AOIs are large and well separated. Since he is not interested in how the eye moves during saccades, a low sampling frequency provides enough information about when participants looked at an object. For Boris, a cheap remote eye tracker attached to a laptop would suffice, but other remote eye trackers may work equally well. In terms of data analysis, Boris needs to decide which AOI each gaze sample is located in. This type of analysis is supported in most commercial software.

Since pupil size changes are quite slow in relation to eye movements, Ingrid, who wanted to study pupil size changes in response to stress, found that a low frequency eye tracker that outputs pupil area or pupil diameter is sufficient. To avoid potential confounds in pupil size estimates from participants moving relative to the eye tracker (Petersch & Dierkes, 2022; Pfeffer et al., 2013), she decided to use a head-boxed eye tracker and put participants in a chin, and forehead rest. Since large gaze angles can lead to apparent pupil size changes in eye-tracker data recorded from VOG eye trackers (Hayes & Petrov, 2016; Gagl, Hawelka, & Hutzler, 2011), she was also considering whether to allow participants to move their gaze freely, or to restrict their gaze to the center of the screen. The reason she avoided buying a head-mounted eye tracker, for instance a pair of glasses, was that she aimed for computer screen-based research, where it seemed more reliable and easier to synchronize the eye tracking data to the visual stimulus presentation using a remote eye tracker. Ingrid thinks using a standard web camera with open-source software may also be sufficient for her research (e.g., Petridis et al., 2013; Wisiecka et al., 2022). However, it should be noted that changes in pupil diameter may not be accurately estimated by current web cameras, as many of them do not use infrared light, which makes it easier to separate the pupil from the background in the captured images (Kaduk et al., 2024). Depending on the type of analysis Ingrid wants to conduct, particular attention should be placed on the pre-processing of the pupil size data (Kret & Sjak-Shie, 2019).

Kim is interested in whether people with a university degree read differently than people without a university degree, and how reading changes with age (elderly vs. adolescents). He finds that much of the reading research with eye tracking has been conducted with head fixed setups (e.g., Rayner, Chace, Slattery, & Ashby, 2006). Common units of analysis in reading research are sentences, words, or even individual characters (Inhoff & Radach, 1998). To be able to tell whether a participant looks at such units, a critical requirement for Kim is to have an eye tracker capable of recording accurate gaze position data. To be able to compute eye-tracking measures in relation to words, he needs to 1) segment words into areas of interests (AOIs), 2) detect fixations from gaze position data recorded from the eye tracker, and 3) map the fixations to the AOIs. He thinks that programming, something he does not have any experience with, is required to perform these steps with sufficient accuracy and in a reasonable amount of time. Fortunately, he finds commercial software that includes this functionality (Niehorster et al., 2025).

In his endeavor to collect eye-movement data from infants to study their cognitive development, Sven requires an eye tracker that allows for easy and quick positioning of the infant and flexible calibration of the eye tracker. Moreover, the eye tracker ideally is able to deal with some movement and/or suboptimal positioning of the infant, such that the eye-tracking data are still usable, i.e., Sven can still infer where and when the infant looks on the computer screen. He found that many remote eye-trackers seem to meet some of the requirements for his research (Hessels et al., 2015b; Niehorster et al., 2018), even though he noted that head-mounted eye trackers, although less common, have also been successfully used to record infant’s gaze (e.g., Franchak et al., 2010). He notes that appropriate methods to restrain the infants (seating options) and position the eye tracker relative to the infants appear to be equally important as the chosen eye tracker type and model (Hessels & Hooge, 2019). Data recorded from infants are often of lower quality than data recorded from adults (Hessels & Hooge, 2019). Therefore, a software requirement may include the option to export the raw data from a recording, to be able to apply custom tools designs for, for instance, fixation classification in noisy data (Hessels et al., 2017).

Kerstin aims to use eye tracking to examine how often and at what moments the striker looks at the ball, goal, and goalkeeper before taking a penalty shot in football." She finds that a head-mounted eye tracker is the only viable option. Since she plans to record moving participants outdoors on a pitch without electricity, she considers robustness to slippage of the eye tracker relative the head (Niehorster et al., 2020b; Onkhar, Dodou, & De Winter, 2024; Hooge et al., 2023), a good battery life, and robustness to weather conditions to be important requirements. After her first pilot analysis, Kerstin realizes that it will be too time consuming to go through the videos manually and note in an Excel-sheet every frame the striker looks at the ball, goal, or goalkeeper. Therefore, she investigates whether automatic processing pipelines can be employed. She finds a range of publicly available video processing and analysis tools that can segment (Kirillov et al., 2023) and label objects (Liu, Tao, Liang, Li, & Chen, 2018b) in a video frame. It then appears possible to automatically map gaze points recorded by the eye tracker to objects like the ball, goal, and goalkeeper in the scene video of the eye tracker. She now considers bringing in a collaborator to help with the programming to avoid spending a substantial amount of time manually coding the scene videos. Finally, Sam is interested in studying covert attention by observing microsaccade directions. His main requirement is that the eye tracker should be able to record microsaccades, that is, saccades with small amplitudes performed during attempted fixation (Rolfs, 2009). Two eye tracker properties have been shown to be particularly important when studying microsaccades: precision and resolution. The former means that the gaze position signal should have a low noise level, and the latter is a measure of the smallest eye rotation an eye tracker can estimate (cf. Poletti and Rucci, 2016; Holmqvist & Blignaut, 2020; Nyström et al., 2023). Typically, this requires the use of high-end head fixed eye trackers. Sam finds that a standard method to detect microsaccades is the algorithm by Engbert and Kliegl (2003), which typically uses data from both eyes, and considers a microsaccade to occur only if overlapping saccades are detected from both eyes. Thus, an eye tracker allowing binocular recordings is required.

For the above personas, we have discussed the minimal requirements for choosing an eye tracker. However, even though an eye tracker fulfills the minimal requirement for a research scenario, is there a reason not to choose an eye tracker with even better specifications? Consider Boris’ research, for instance, that is not very demanding in terms of technical specification of the eye tracker (accuracy, precision, and sampling frequency). However, in his next study, perhaps Boris wants to run an experiment that demands better technical specification. In that case, Boris may be well served by choosing an eye tracker with better specifications already from the beginning.

To conclude, choosing an eye tracker is more than looking at the technical specifications of an eye tracker. Carefully considering the requirement of the research is often helpful, along with testing the eye tracker, considering the whole eye-tracker setup, as well as talking to other researchers who have experience from eye-tracking research in similar fields. We hope that this article will help researchers ask the right questions and make them more confident in their choice of an eye tracker.

## Footnotes

1 https://www.blackboxtoolkit.com

2 https://cedrus.com/stimtracker/index.ht/

## Declarations

### Open Practices Statement

There are no data or code associated with the present article.
